# Supplementary material for: PMSFF: Improved Protein Binding Residues Prediction through Multi-Scale Sequence-Based Feature Fusion Strategy
Source: Biomolecules. 2024 Sep 27;14(10):1220. doi: 10.3390/biom14101220 (PMC11506650; doi:10.3390/biom14101220)
Supplement: Supplementary file 1 [file biomolecules-14-01220-s001.zip › biomolecules-3184837-supplementary.pdf]

Supplementary Material

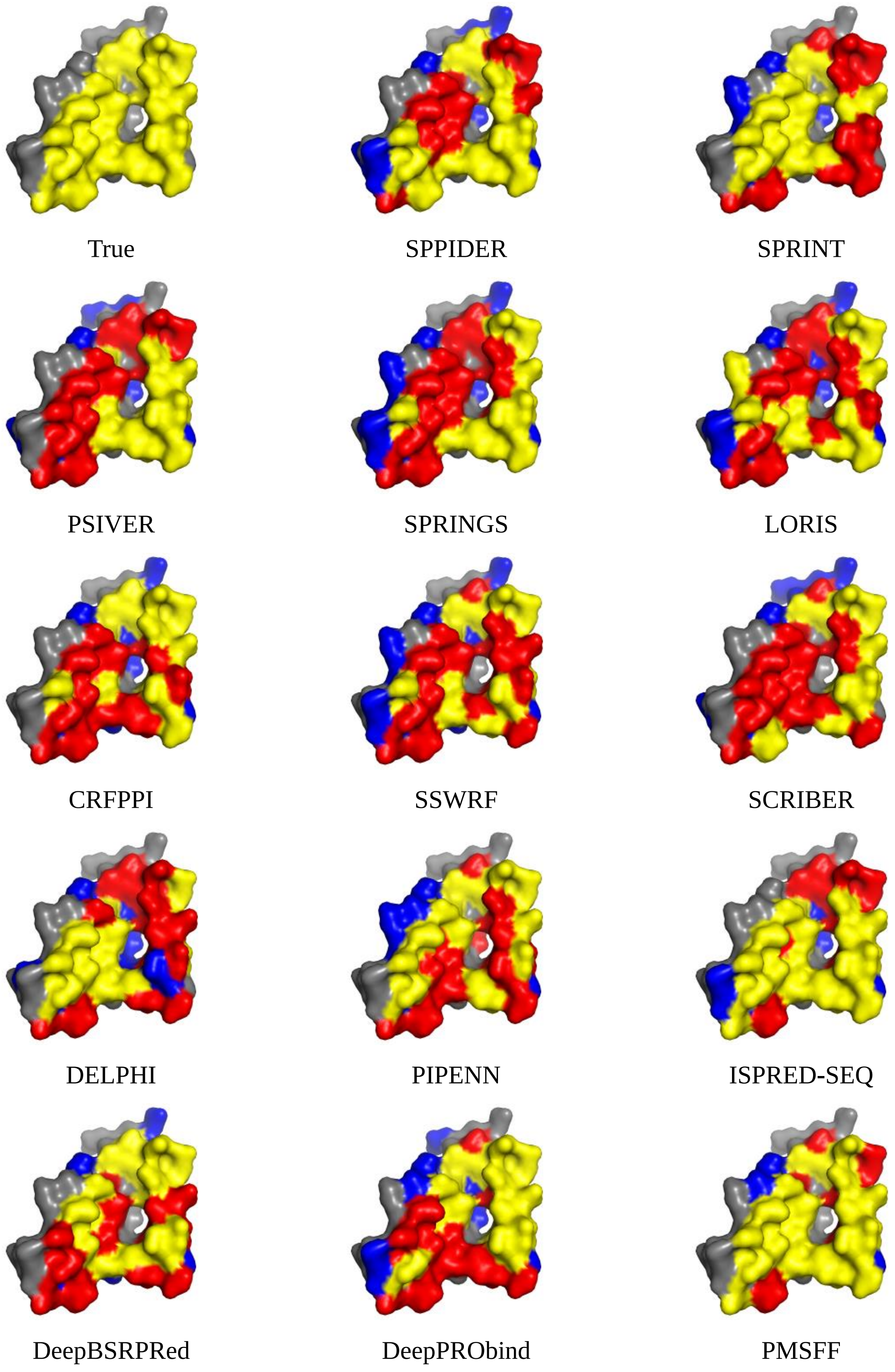

Fig. S1 Interaction sites of a representative protein in the NSP448 set (PDB ID: 3ZCB, Chain: B). TP predictions are in yellow, FN predictions are in red, FP predictions are in blue and the background grey represents TN predictions. The threshold of each method is determined by making the number of predicted interaction residues equals that of actual interaction residues.
